# Detection of antigens and anti‐*Toxocara canis* antibodies in children with different asthma severities

**DOI:** 10.1002/iid3.403

**Published:** 2021-02-08

**Authors:** Bautista‐García Sandra Guadalupe, Martínez‐Gordillo Mario Noé, Peralta‐Abarca Gustavo Esteban, González‐Bobadilla Norma Yvett, Clavijo‐Sánchez Karina, Chávez‐Zea Alma Leticia, Hernández‐Saavedra Alan Eduardo, Huerta‐López José Guadalupe, Pedroza‐Meléndez Álvaro, González‐Garay Alejandro Gabriel, Ponce‐Macotela Martha

**Affiliations:** ^1^ Servicio de Inmunología y Alergia, Departamento de Consulta Externa de Pediatría Instituto Nacional de Pediatría (INP) Ciudad de México México; ^2^ Laboratorio de Parasitología Experimental, Subdirección de Medicina Experimental Instituto Nacional de Pediatría (INP) Ciudad de México México; ^3^ Departamento de Biología, Facultad de Ciencias Universidad Nacional Autónoma de México Ciudad de México México; ^4^ Coordinación del Servicio Social, Facultad de Medicina Universidad Nacional Autónoma de México Ciudad de México México; ^5^ Departamento de Metodología de Investigación Instituto Nacional de Pediatría (INP) Ciudad de México México

**Keywords:** active larva *migrans*, asthma, children, chronic infection, recent infection, seroprevalence, *T. canis* secretory‐excretory antigens, *Toxocara canis*

## Abstract

**Introduction:**

*Toxocara canis* can produce or exacerbate asthma, and the detection of anti‐*T. canis* immunoglobulin G (IgG) does not discriminate between recent infection or active larva *migrans*. In this study, we searched for *T. canis* third‐stage larval antigens (L_3_TES) and anti‐*T. canis* antibodies in children with different severities of asthma, controlled or uncontrolled.

**Methods:**

A total of 145 patients with asthma who were previously diagnosed using the Global Initiative for Asthma guidelines were included. The asthma control was evaluated with the Asthma Control Questionnaire (ACQ). Enzyme‐linked immunosorbent assay was performed for the detection of L_3_TES; IgG was detected using sera preadsorbed with *Ascaris* antigens (native kit), and a commercial kit (IgG) was used as the gold standard.

**Results:**

L_3_TES was found in 2 patients (1.37%). One had L_3_TES and anti‐*T. canis* IgG, suggesting active larva *migrans*. In the other patient, only L_3_TES was detected, likely because an infection had begun. The seroprevalence with the commercial kit and native kit was 6.2% and 17.93%, respectively. There was no significant association among asthma severity, ACQ and *T. canis* seroprevalence (*p* > .05).

**Conclusion:**

It is possible to detect L_3_TES in patients with asthma. Two complementary techniques that can determine the infection status with *T. canis* and rule out cross‐reactions involve the detection of L_3_TES and IgG using sera preadsorbed with *Ascaris* antigen. There was no significant association among asthma severity, ACQ and *T. canis* seroprevalence.

## Graphical abstract

We performed two assays aimed to detect antigens from Toxocara third‐stage larvae, and to detect anti‐T‐canis immunoglobulin G in children with asthma, to infer the status of the infection and its impact on the asthma severity. We detected patients with Toxocara antigens (recent infection); antigens and antibodies (active larva migrans) and only antibodies (immunological memory). There was no significant association among asthma severity and detection of antigens or antibodies against T. canis.

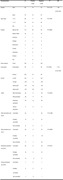

## 1 INTRODUCTION

Asthma is a global health problem that is increasing in children,^1^ and one instrument used to determine the controlling this pathology is the Asthma Control Questionnaire (ACQ).^2^ Environmental exposure to *Toxocara canis* may be a risk factor for asthma, or parasitic infections may increase pulmonary symptoms in asthmatic patients.^3^, ^4^ *T. canis* is a zoonotic nematode, and canids are the definitive hosts. *T. canis* eggs that exit in the feces of canids are not infectious and need to be in the environment for 9–15 days for the third‐stage larva to develop inside the egg to be infective.^5^ Humans, other mammals and birds are paratenic hosts (i.e., the larva does not develop), and this parasite remains as a third‐stage larva.^6^

Children are more susceptible to infection with *T. canis* due to playing habits or from soil consumption; they can also be infected with this parasite by consuming contaminated vegetables and by ingesting insufficiently cooked liver or meat from other mammals or infected birds.^7^, ^8^, ^9^ *T. canis* in humans causes ocular larva *migrans* (OLM), cerebral toxocariasis, visceral larva *migrans* (VLM) and covert toxocariasis (COT). In the latter two cases, children may be asymptomatic or may have asthma symptoms, among other symptoms.^10^ Because the larvae of *Toxocara* causes a granulomatous response that puts the function of the affected organs at risk, it is necessary to detect patients with recent infection to provide timely treatment and thus avoid complications.

The diagnosis of larva *migrans* is immunological through immunoglobulin G (IgG) detection, and the seroprevalence is lower in developed countries than in developing countries.^11^, ^12^, ^13^, ^14^, ^15^ In Mexico, it ranges from 12% to 26%.^16^

Some reports show that the seroprevalence of *T. canis* is higher in patients with asthma than in patients without asthma: 6% versus 2% in Iran,^17^ 29% versus 10% in Sri Lanka,^18^ 57% versus 52.8% in Argentina,^19^ and 30.8% versus 19.7% in Mexico.^20^ However, there may be cross‐reactions with other nematodes, and no distinction can be made between recent infection or chronic infection.

Due to *Ascaris lumbricoides is the most frequently reported soil‐transmitted helminth in developing countries, and its biological cycle involves its migration through the respiratory* system causing Löeffler syndrome;^21^ it is necessary to rule out this parasitic disease in patients with suspected VLM produced by *Toxocara canis*.

To avoid cross‐reactions between *Ascaris* and *Toxocara*, the use of competitive enzyme‐linked immunosorbent assay (ELISA) has been proposed.

Albendazole is indicated in both parasitic diseases, but owing to *Toxocara* larvae can affect the eye or the central nervous system, then it will be necessary to consider an antiinflammatory or surgical treatment.

Few studies have shown the possibility of detecting L_3_TES in the peripheral blood of patients with anti‐*T*. *canis* IgG.^22^, ^23^, ^24^, ^25^ In a longitudinal study of the host‐parasite interaction in a murine model, we monitored the production of antibodies and the detection of L_3_TES. We proposed that the detection of L_3_TES could indicate a recent infection and that the detection of L_3_TES and IgG could indicate an active infection.^26^

With this background in mind, in this study, we set out to detect L_3_TES and anti‐*T. canis* immunoglobulins in pediatric asthmatic patients to identify recent infection or active larva *migrans* and to demonstrate whether parasitic infection impacts asthma severity.

## 2 METHODS

### 2.1 Study design and participants

This study was observational, cross‐sectional, prospective, and analytical. The inclusion criteria were as follows: children 6–18 years of age with a diagnosis of asthma made based on clinical criteria and spirometry, who attended outpatient consultation at the Allergy Clinic of the National Institute of Pediatrics (Instituto Nacional de Pediatría, INP) from 2016 to 2018. This study was approved by the Research and Ethics Committees of the INP with registration number 070/2015.

The exclusion criteria were children with other pulmonary conditions, oncological diseases, or immunodeficiencies, or those who had airway infections of at least 1 week of evolution. Letters of consent or informed assent were obtained.

### 2.2 Global Initiative for Asthma and ACQ questionnaires, spirometry, hematic biometry, and stool exams

Parents or patients answered the Global Initiative for Asthma questionnaire and ACQ, and the latter was kindly donated to the National Institute of Pediatrics by Dr. Juniper EF. Spirometry and the reversibility test were performed using the standard technique, with SpiroUSB equipment (CareFusion). Two tubes with 3 ml of blood were obtained, one for hematic biometry and the other for immunological tests. To rule out other soil‐transmitted helminth infections, such as ascariasis, strongyloidiasis and trichocephalosis, three stool samples from the patients were requested, and direct exams and the Faust flotation technique were performed.

### 2.3 Immunological techniques

ELISAs were used for the detection of L_3_TES.^27^ IgG detection was performed with and without sera preadsorbed with *Ascaris suum* antigen (native kit, performed with antigen and antibodies obtained in our laboratory). A commercial kit (nonnative) that detects IgG (AccuDiag) was used as the gold standard.

### 2.4 Sandwich ELISA for the detection of L_3_TES

Polystyrene plates (3590; Costar) were sensitized with 5.0 µg/ml polyclonal antibody overnight at 4°C. Subsequently, nonspecific sites were blocked. To break the immune complexes, the sera were treated with 0.1M Ethylenediaminetetraacetic acid pH 7.5 (1:1), boiled for 5 min and centrifuged at 12,000 rpm/10 min. The treated serum was added to the wells, and the plate was incubated for 2 h at 37°C. A total of 10 µg/ml of the antibody obtained from the 1E4B7B12 hybridoma was added and incubated for 2 h at 37°C. Secondary antibody anti‐mouse IgG conjugated with horseradish peroxidase (1:500) was added, and the plate was incubated for 2 h at 37°C. The chromogen was added, the reaction was stopped with 2N sulfuric acid, and the plates were read at 490 nm in a spectrophotometer (Multiskan Go; Thermo Fisher Scientific).^26^, ^27^

### 2.5 Indirect ELISA for the detection of antibodies

For the detection of IgG, a commercial (nonnative) kit (AccuDiag) was used according to the manufacturer's instructions.

For ELISA (native), the wells were sensitized with 0.5 μg/ml of L_3_TES and incubated overnight at 4°C. The wells were blocked, and then 1:64 diluted serum was added and incubated for 1 h/37°C. Secondary antibody anti‐human IgG conjugated with horseradish peroxidase (1:1000) was added (Merck), and the wells were incubated for 1 h at 37°C. Chromogen was added, and the wells were incubated in the dark at room temperature for 15 min. In all cases, the corresponding washes were performed. The reaction was stopped with 2N sulfuric acid, and the absorbance was measured in an ELISA reader at 490 nm (Multiskan Go; Thermo Fisher Scientific).^26^

Competitive ELISA was performed to rule out cross‐reactions with *Ascaris*. Batch of sera (1:64) was preadsorbed with *Ascaris suum* antigens (As‐Ag) (1 μg/ml) for 2 h/37°C. Subsequently, the methodology described for the detection of IgG was followed.

The *Toxocara*‐specific IgG avidity was performed following the methodology for the detection of IgG. However, in this case, one of the plates was washed with urea at 7.5M and the other with phosphate‐buffered saline (PBS)‐Tween 20. The avidity index was calculated based on the absorbance at 490 nm of the urea‐treated plate between the absorbance of the plate treated with PBS‐Tween 20 and multiplied by 100. Values less than 50% indicated low IgG avidity and, therefore, recent infection.^28^

In all cases, three assays were performed in duplicate.

### 2.6 Statistical analyses

The categorical and numerical variables are presented as frequencies. For the analysis of *T. canis* seropositivity, 2 × 2 contingency tables, diagnostic test evaluation, and receiver operating characteristic (ROC) curve analysis were performed. Bivariate analysis was performed to evaluate the association between *T. canis* seropositivity and asthma severity using the Mantel‐Haenszel *χ* ^2^ test (*p* < .05 was considered significant).

## 3 RESULTS

A total of 145 asthmatic patients were analyzed. The patients' age, gender, and place of residence are shown in Table 1. L_3_TES was detected in two patients (1.4%) who were both from Mexico City. One 8‐year‐old male patient had mild partially controlled persistent asthma, and 0.1 μg/ml of L_3_TES was detected. There was no eosinophilia, and the only finding on the stool tests was *Blastocystis hominis*. The other 7‐year‐old patient had uncontrolled moderate persistent asthma, and 8.96 μg/ml of L_3_TES and anti‐*T. canis* IgG was detected, without eosinophilia or other parasites (Table 1).

**Table 1:** Seroprevalence of *Toxocara canis* in children with asthma

Characteristics	*N*	Positive *n* (%)	Negative *n* (%)	*χ* ^2^	Odds ratio
Gender					
Girls	66	14	52	*p* = .1733	1.49 (0.63–3.58)
Boys	79	12	67		
Age range					
5–10	69	11	58	*p* = .7564	
11–17	65	13	52		
ND	11	2	9		
Estates					
Mexico City	113	20	93	*p* = .6899	
Mexico State	18	3	15		
Guerrero	4	1	3		
Hidalgo	3	0	3		
Michoacan	3	1	2		
Puebla	1	0	1		
Queretaro	1	0	1		
Sinaloa	1	1	0		
ND	1	0	1		
Stool analysis					
Faust	145	0	145		
Eosinophils					
Eosinophilia	36	7	29	*p* = .7848	1.143 (0.43–2.99)
Normal	109	19	90		
ELISA^a^					
L_3_TES^b^	145	2	143		
Kit IgG^c^	145	9	136		
Native IgG^d^	145	25	120		
Native IgG^e^	145	26	119		
GINA^f^					
Mild intermittent	44	5	39	*p* = .5874	
Mild persistent	48	11	37		
Moderate persistent	49	10	39		
Severe persistent	4	0	4		
Mild intermittent and ACQ^g^					
Controlled	23	1	22	*p* = .1098	
Partially controlled	15	2	13		
Not controlled	6	2	4		
Mild persistent and ACQ					
Controlled	20	5	15	*p* = .2906	
Partially controlled	17	5	12^h^		
Not controlled	11	1	10		
Moderate persistent and ACQ					
Controlled	24	6	18	*p* = .9151	
Partially controlled	12	0	12		
Not controlled	13	4^i^	9		
Severe persistent and ACQ					
Controlled	1	0	1		
Partially controlled	2	0	2		
Not controlled	1	0	1		

Abbreviations: ACQ, Asthma Control Questionnaire; ELISA, enzyme‐linked immunosorbent assay; IgG, enzyme‐linked immunosorbent assay.

a Enzyme‐linked‐immunosorbent assay.

b *T. canis* third‐stage larval antigens.

c Commercial kit (IgG).

d Native kit (sera without preadsorbed with Ascaris suum antigens).

e Native kit (sera preadsorbed with Ascaris suum antigens).

f Global Initiative for Asthma.

g Asthma Control Questionnaire.

h One patient with L_3_TES and without IgG.

i One patient with L_3_TES and IgG.

The seroprevalence obtained with the commercial kit (nonnative) for IgG detection was 6.2%; whilst with the (native) kit without preadsorbed As‐Ag was 17.24%; and with the (native) kit with preadsorbed As‐Ag was 17.93% (Table 1).

In the ELISA performed with sera preadsorbed with As‐Ag, negativization was found in three sera that had been positive on the ELISA without adsorption, and four samples that were initially negative after adsorption became positive.

According to the results of the ELISAs (L_3_TES and IgG with sera preadsorbed with Ag‐As), the patient with L_3_TES likely had a recent infection. The patient who had both L_3_TES and IgG likely had active larva *migrans*, and the other 25 patients (IgG) had immunological memory or a past infection.

No significant association was found between the seroprevalence to *T. canis* and gender, age, or place of residence (*p* > .05). No association was found with asthma severity, asthma control or eosinophilia (Table 1).

The sensitivity, specificity, and predictive values of the different tests are shown in Table 2. To discriminate among the commercial (nonnative) kit and the native kit (sera preadsorbed with As‐Ag), and both natives with and without preadsorbed sera for IgG detection, a ROC curve was developed. In the first case, an area under the curve of 0.70 was found, and both increased to 0.91.

**Table 2:** Sensitivity and specificity of the different tests for the detection of *Toxocara canis* in patients with asthma

**ELISA**	**Sensitivity (95% CI)**	**Specificity (95% CI)**	**PPV (95% CI)**	**NPV (95% CI)**	**Diagnostic accuracy (95% CI)**	**AUC (IC95%)**
IgG commercial kit vs. L_3_TESe^a^	11.11 (1.98–43.5)	99.26 (95.95–99.87)	50 (9.45–90.55)	94.41 (89.35–97.14)	93.79 (88.63–96.7)	0.55 (0.48–0.71)
IgG commercial kit vs. IgG native kit^b^	55.56 (26.66–81.12)	84.56 (77.55–89.67)	19.23 (8.50–37.88)	96.64 (91.68–98.69)	82.76 (75.79–88.04)	0.70 (0.52–0.85)
IgG native kit^b^ vs. IgG native kit^c^	84.62 (66.47–93.85)	97.48 (92.85–99.14)	88 (70.04–95.83)	96.67 (91.74–98.7)	95.17 (90.37–97.64)	0.91 (0.79–0.96)
IgG native kit^b^ vs eosinophilia	26.92 (13.7–46.08)	75.63 (67.19–82.46)	19.44 (9.75–35.03)	82.57 (74.37–88.55)	66.9 (58.89–74.03)	0.51 (0.40–0.64)

Abbreviations: AUC, area under the curve; CI, confidence interval; ELISA, enzyme‐linked immunosorbent assay; NPV, negative predictive value; PPV, positive predictive value.

^a^ *T. canis* third‐stage larval antigens.

^b^ Native Kit (sera preadsorbed with Ascaris suum antigens).

^c^ Native Kit (sera without preadsorbed with Ascaris suum antigens).

In 21 samples (80.76%), the avidity was greater than 50%. In five samples (19.23%), the avidity was less than 50%. The sample from the patient with L_3_TES and IgG had an avidity of 91.8%.

The serial stool exams of 3 alternating days were negative for helminths.

## 4 DISCUSSION

Asthma is a heterogeneous disease in which predisposing and triggering factors are involved.^1^ *Toxocara canis* can be a trigger factor for asthma. Because *Toxocara* larvae in paratenic hosts can live for several years in the hypobiotic phase^6^ and because the presence of anti‐*T. canis* IgG does not discriminate between recent infection and active infection,^11^ it is necessary to implement other strategies to determine the course of the disease.

In this study, we showed that it is possible to detect L_3_TES in patients with asthma. The results are relevant because the presence of L_3_TES suggests that the patients had live *Toxocara* larvae; one of them was probably recently infected because only L_3_TES was found. The other patient had already mounted a humoral immune response in the presence of the parasite because anti‐*T. canis* IgG and L_3_TES were detected; this patient perhaps had active larva *migrans*, as previously suggested.^26^

The results are important because asthmatic patients with anti‐*T. canis* IgG and L_3_TES should receive antiparasitic treatment to avoid complications; but these patients should be monitored because they are receiving inhaled corticosteroids.

There are few but interesting reports that demonstrate the detection of L_3_TES in patients with anti‐*T. canis* IgG. Robertson et al.^22^ detected L_3_TES in two of three patients with VLM, in one patient with OLM and in 10 of 23 children with probable filariasis or malaria. Guillespie et al.^25^ detected L_3_TES in 19 of 28 patients with positive IgG and VLM, in one of 10 patients with inactive VLM and in two of seven patients with OML. Luo et al.^23^ detected L_3_TES in 39.5% of 43 sera samples with positive IgG, and Ishiyama et al.^24^ detected L_3_TES in five of nine patients with symptomatology and positive IgG. In our case, we detected L_3_TES in patients diagnosed with asthma, but there was no significant difference between L_3_TES detection and asthma severity (*p* > .05); this finding was likely because there were few patients in whom L_3_TES was detected.

With the methodology we used, L_3_TES can be detected from 470 pg/ml.^27^ Other authors have reported detection values ranging from 0.004 to 0.078 µg/ml.^23^, ^24^ In a model of L_3_TES release kinetics in mice intraperitoneally injected with 100 larvae, 1.5 µg/ml of L_3_TES was found at Day 140 postinfection.^26^ In this study, one of the patients had 5.6 times more L_3_TES than the one detected in mice, suggesting that the detection of L_3_TES can range from picograms to several micrograms, and further studies will be needed to determine if the concentration of L_3_TES influences in the disease manifestation.

With respect to IgG detection in patients with varying severities of asthma, we used a commercial kit (nonnative) as the gold standard and a native kit, with and without sera preadsorption with As‐Ag, with the purpose of detecting cross‐reactions with *Ascaris*.^20^ Greater seroprevalence was found with the native kit than with the commercial kit (nonnative), perhaps because we sensitized the plate with a mixture of L_3_TES, possibly increasing the number of epitopes recognized. A disadvantage is the labor‐intensiveness of obtaining L_3_TES.

After the 145 sera were adsorbed with As‐Ag, it was found that three sera that had been positive for *T. canis* subsequently became negative; these results are interesting because three sera were eliminated by cross‐reaction. Although the stool tests in triplicate were negative for other helminths, we cannot rule out infection with another nematode in the process of migration.

On the other hand, it is also important to note that four sera that were negative became positive after adsorption with As‐Ag, likely due to the release of *T. canis* antibodies after adsorption. These results show that indirect ELISA with sera preadsorbed with As‐Ag rules out cross‐reactions and can be easier and faster than Western blot.

The migration of *T. canis* larvae in the respiratory system can produce or exacerbate asthma.^3^, ^19^, ^29^ In this study, none of the four children with severe asthma (controlled, partially controlled, or uncontrolled) were seropositive for *T. canis*. We found no association between asthma severity and IgG seropositivity (*p* > .05), nor did we find an association with asthma control (ACQ) and seropositivity (*p* > .05). Other authors have reported greater seropositivity in asthmatic patients than in people without asthma: Muñoz‐Guzmán et al.^20^ reported a seroprevalence of 30.8% in Mexican pediatric patients with asthma. Fernando et al.^18^ detected a 29% seroprevalence in 100 asthmatic patients from Sri Lanka.

It has been documented that a high avidity index corresponds to past infection.^28^ We found that 80.7% of the samples had high avidity, and this was correlated with negative L_3_TES. The patient with L_3_TES and IgG is noteworthy because the avidity index was high (91.8%), but this finding may indicate that the child infected still had the alive larvae, as has been suggested.^29^

Given the high sensitivity and specificity between the ELISA with and without adsorption with As‐Ag, coupled with the detection of cross‐reactions, ELISA should only be performed with sera adsorbed with As‐Ag to reduce costs. If we add ELISA for detecting L_3_TES, with these two tests, patients with recent infection, active larva *migrans*, and chronic infection or immune memory can be detected.

IgG detection can be used for screening, but the detection of L_3_TES is necessary for determining the status of the infection.

In conclusion, it is possible to detect L_3_TES in patients with asthma. There was no significant association between *T. canis* seropositivity and asthma severity. No significant association was found with asthma control according to the ACQ. Two complementary techniques that allow the determination of *T. canis* infection status and rule out cross‐reactions involve detection of L_3_TES and IgG with preadsorbed As‐Ag.

## Data Availability

The data that supports the findings of this study are available in the Supporting Information material of this article.
